# Implication of Hyperhomocysteinemia in Blood Retinal Barrier (BRB) Dysfunction

**DOI:** 10.3390/biom10081119

**Published:** 2020-07-29

**Authors:** Amany Tawfik, Yara A. Samra, Nehal M. Elsherbiny, Mohamed Al-Shabrawey

**Affiliations:** 1 Department of Oral Biology and Diagnostic Sciences, Dental College of Georgia, Augusta University, Augusta, GA 30912, USA; ysamra@augusta.edu (Y.A.S.); drnehal@mans.edu.eg (N.M.E.); malshabrawey@augusta.edu (M.A.-S.); 2 James and Jean Culver Vision Discovery Institute, MCG, Augusta University, Augusta, GA 30912, USA; 3 Department of Cellular Biology and Anatomy, Medical College of Georgia (MCG), Augusta University, Augusta, GA 30912, USA; 4 Department of Ophthalmology, MCG, Augusta University, Augusta, GA 30912, USA; 5 Department of Biochemistry, Faculty of Pharmacy, Mansoura University, Mansoura 35516, Egypt; 6 Department of Anatomy, Faculty of Medicine, Mansoura University, Mansoura 35516, Egypt

**Keywords:** hyperhomocysteinemia, blood retinal barrier, blood brain barrier, dysfunction, mechanisms

## Abstract

Elevated plasma homocysteine (Hcy) level, known as hyperhomocysteinemia (HHcy) has been linked to different systemic and neurological diseases, well-known as a risk factor for systemic atherosclerosis and cardiovascular disease (CVD) and has been identified as a risk factor for several ocular disorders, such as diabetic retinopathy (DR) and age-related macular degeneration (AMD). Different mechanisms have been proposed to explain HHcy-induced visual dysfunction, including oxidative stress, upregulation of inflammatory mediators, retinal ganglion cell apoptosis, and extracellular matrix remodeling. Our previous studies using in vivo and in vitro models of HHcy have demonstrated that Hcy impairs the function of both inner and outer blood retinal barrier (BRB). Dysfunction of BRB is a hallmark of vision loss in DR and AMD. Our findings highlighted oxidative stress, ER stress, inflammation, and epigenetic modifications as possible mechanisms of HHcy-induced BRB dysfunction. In addition, we recently reported HHcy-induced brain inflammation as a mechanism of blood–brain barrier (BBB) dysfunction and pathogenesis of Alzheimer’s disease (AD). Moreover, we are currently investigating the activation of glutamate receptor *N*-methyl-d-aspartate receptor (NMDAR) as the molecular mechanism for HHcy-induced BRB dysfunction. This review focuses on the studied effects of HHcy on BRB and the controversial role of HHcy in the pathogenesis of aging neurological diseases such as DR, AMD, and AD. We also highlight the possible mechanisms for such deleterious effects of HHcy.

## 1. Formation and Metabolism of Homocysteine 

**Figure 1:**
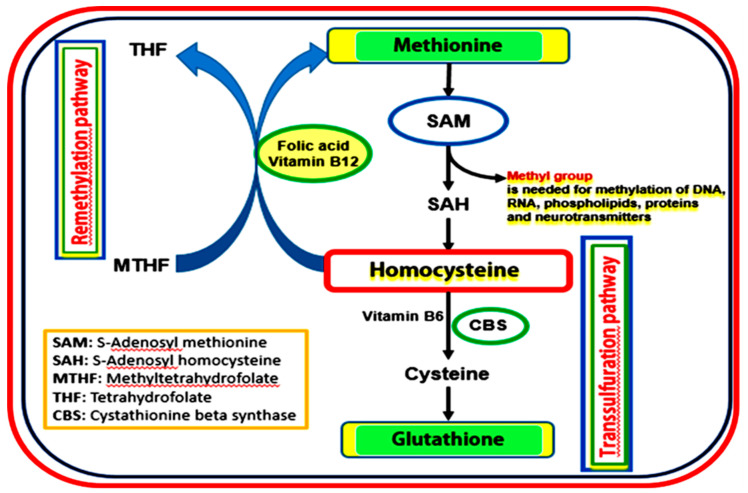
Pathways for homocysteine metabolism, showing the two main pathways for Hcy metabolism, the remethylation, and transsulfuration pathways.

Homocysteine (Hcy) is a sulfur-containing amino acid that is produced in the body by demethylation of the essential amino acid methionine, which is found mainly in red meat and dairy products. Hcy is normally present in the plasma in low concentration (12–15 µmol/L). Nevertheless abnormal increase of Hcy level is harmful and is identified as hyperhomocysteinemia (HHcy) [1] which is prevalent in the human population (5–12%). As shown in (Figure 1), there are two main pathways for Hcy metabolism. The first pathway (Remethylation pathway) is the main pathway for Hcy metabolism and occurs when methionine level is low. Hcy receives a methyl group to be converted to methionine via remethylation reaction in presence of vitamins cofactors, vitamin B12 and methyl tetrahydrofolate (active form of folic acid, vitamin B9). Methionine is a precursor of S-adenosyl-l-methionine (SAM), which is an important methyl donor needed for many biological reactions in the body such as; transmethylation reactions of DNA, RNA, proteins, and lipids, the biosynthesis of polyamines, biotin, nicotianamine, and ethylene [2]. The second pathway for Hcy metabolism, transsulfuration pathway is activated in the presence of excess methionine and Hcy is converted to cystathionine and subsequently to cysteine (precursor of antioxidant glutathione) by cystathione-β-synthase (CBS) enzyme and vitamin B6 as a cofactor. Changes in the concentration of methionine in dietary intake or deficiencies in enzymes and vitamin cofactors needed for Hcy metabolism such as folic acid, vitamin B12, and vitamin B6 affect Hcy metabolism and lead to the accumulation of Hcy in the body producing harmful effects [3,4]. Indeed, various studies linked HHCy to vitamin B deficiency in patients with cardiovascular diseases [5], chronic kidney disease [6], Alzheimer’s Disease [7], cerebral infarction [8], diabetic complications [9,10], and bone disease [11]. In addition to the reported Hcy-lowering efficacy with B vitamins, omega-3 polyunsaturated fatty acids (n-3 PUFAs) have also gained attention as a class of nutrients with a possible protective effect on HHcy-induced cardiovascular injury [12]. In this context, Huang et al. performed a meta-analysis of eleven randomized, placebo-controlled trials that focused on the effect of n-3 PUFAs on Hcy levels. Interestingly, the analysis suggested that ω-3 PUFA supplementation can decrease plasma Hcy levels [13]. Whether the interaction between n-3 PUFAs and Hcy is independent and/or synergistic with the B vitamins status remains to be elucidated. 

Moreover, Hcy level is markedly increased in people who have genetic defects in the Hcy metabolism. Homocystinuria is an inborn error of metabolism caused by a disruption of the remethylation or transsulfuration pathways of Hcy metabolism. The three most frequent genetic causes of homocystinuria are severe MTHFR deficiency, CBS deficiency which is called classical homocystinuria and methylmalonic aciduria with homocystinuria cblC type [14,15,16]. In the remethylation pathway, deficiency of methylenetetrahydrofolate reductase (MTHFR) is the most common genetic abnormality. MTHFR is an enzyme that catalyzes the conversion of 5, 10-methylenetetrahydrofolate to 5-methyltetrahydrofolate, the major circulatory form of folate. 5-Methyltetrahydrofolate is the carbon donor for Hcy remethylation to methionine, a reaction catalyzed by methionine synthase [17]. Moreover, inborn error of cobalamin (Cbl), vitamin B (12) metabolism resulting in methylmalonic aciduria with homocystinuria cblC type can occur. While in the transsulfuration pathway, genetic mutation in CBS gene leading to classical homocystinuria is the frequent genetic abnormality [18].

## 2. Structure of the Retina

**Figure 2:**
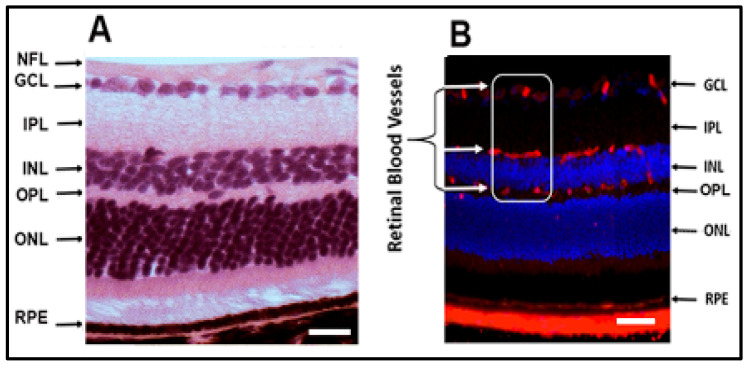
(**A**) Retinal section, isolated from mice eye, stained with hematoxylin and eosin demonstrating different retinal layers as observed by microscopic examination from inside to outside are; ganglion cell (GC), inner plexiform (IPL), inner nuclear (INL), outer plexiform (OPL), outer nuclear (ONL), and retinal pigment epithelium (RPE). (**B**) Immunostaining of retinal section isolated from mice eye, stained for isolectin-B4 (vascular marker, red) and DAPI (nuclear marker, blue), showing that retinal blood vessels (inside the white box) are located in three different layers, the inner retinal layers (the nerve fiber and ganglion cell layer, and plexiform layers). Scale bar is 50 µm.

Retina is a light-sensitive layer of nerve tissue coating the inner surface of the eye. The retina creates an image from the light that is received from the cornea and is focused by the crystalline lens. The image projected on retinal surface is transformed into nerve impulses to be sent to the brain. The retina is a highly metabolically active organ with a complex structure and is a part of the central nervous system. Macroscopically, the retina is made of several layers, from inside to outside layers are; nerve fiber (NF), ganglion cell (GC), inner plexiform (IPL), inner nuclear (INL), outer plexiform (OPL), outer nuclear (ONL), and RPE [19] (Figure 2A). Retinal vessels are localized in the inner neural retina, where they are distributed in the nerve fiber layer and the plexiform layers [19] (Figure 2B). Anteriorly, the retina is related to avascular transparent media formed of the vitreous, lens, and cornea. Abnormal development of blood vessels in the transparent vitreous causes interruption of light and vision deterioration such as in DR. 

The retinal pigment epithelium (RPE) is arranged as a single layer of densely packed cells containing a large amount of pigment. RPE ensures selective input of substances from the blood capillaries of the choroid into the retina and plays an important role in maintaining the outer blood retinal barrier. The photoreceptors (rods and cones) are light-sensitive cells and are considered to be the first neuronal cells of the retina. The photoreceptor layer lacks blood vessels and receives its nutrition by diffusion from choroidal blood vessels. While, the outer most layer of the retina contains nerve cells called ganglion cells, which have axons forming the optic nerve that transmits optic messages to the brain. Additionally, the optic nerve contains incoming blood vessels that open into the retina to vascularize the retinal layers and neurons [19]. The macula is the central and the most important part of the retina. The macula contains numerous cones photo receptors and is responsible for central vision that allows good vision in daylight [19]. Diseases in the macula may result in losing the central vision such as in AMD. 

## 3. Effect of Homocysteine on Retinal Vasculature and Blood Retinal Barrier (BRB)

**Figure 3:**
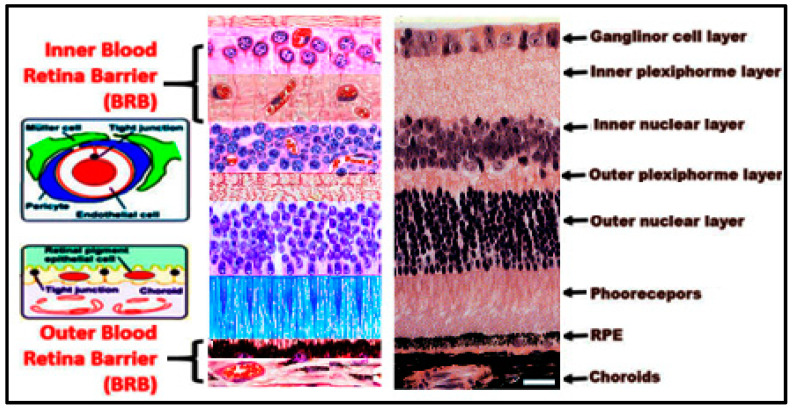
Schematic figure showing the blood retinal barrier arrangement and the components of both the inner and outer blood retinal barrier. The blood retinal barrier has two components, the inner and outer components (inner blood retinal barrier (BRB) and outer BRB). The inner BRB is formed by tight junctions between vascular endothelial, which is surrounded by glial cells, muller cells, and pericytes cells. The outer blood retinal barrier is formed by tight junctions between retinal pigment epithelial cells (RPE), which rests on bruch’s membrane separating RPE from the fenestrated choriocapillaries. Scale bare is 20 µm.

An intact BRB is critical for retinal structural and functional integrity. Vision is adversely affected in clinical conditions where there is breakdown of BRB such as DR [20,21] and AMD [22]. Similar to the BBB, the BRB controls fluid and nutrients molecular movement between ocular vascular beds and retinal tissues and prevents leakage of macromolecules and potentially harmful agents into the retina. BRB is divided into two separate regions: the inner BRB near the vitreous body and the outer BRB near the choroids [23,24]. Inner and outer components of the BRB play a vital role in preserving normal retinal microvascular homeostasis. The inner BRB is formed by tight junctions (TJs) between RECs, which rest on a basal lamina separating them from pericytes and covered by foot processes of astrocytes and Müller cells [20,25]. While the outer BRB is formed by TJs between retinal pigment epithelial (RPE) cells resting on Bruch’s membrane, which separates RPE from the underlying choriocapillaris [26] (Figure 3). DR is initiated by an alteration of the inner BRB, while AMD is characterized by alteration of outer BRB. 

Elevated Hcy disrupts inner [27,28] and outer [29] BRB integrity. Therefore, HHcy increases the BRB permeability and disrupts the structural and functional integrity of the retina. Our previous studies using in vivo and in vitro models of HHcy have shown that HHcy is associated with altered retinal vasculature as evidenced by retinal ischemia, neovascularization, vascular leakage, and a deficient blood–retinal barrier [27,28,29]. Furthermore, we reported altered components of BRB with diminished tight junction proteins (ZO-I, occludin, and claudin-5), pericyte degeneration, glia cells activation [27,28], alteration of retinal pigmented epithelial cell structure, phagocytic and barrier functions [29], induction of neovascularization both in inner retina [27,28], and outer retina with the development of choroidal neovascularization (CNV) [29]. In vivo, two animal models of HHcy were used; the first model was a genetic model, in which one of Hcy clearing enzyme via the transsulfuration pathway is knocked down (CBS deficient mice). Heterozygous (*cbs*^+/−^) mice with one CBS copy have about 4 to 7 fold increase in plasma Hcy and were used to study the effect of mild/moderate HHcy on retinal vasculature and BRB, while the homozygous (*cbs*^−/−^) mice have no copies of cbs with about 30-fold increase in plasma Hcy and represent severe HHcy with severe retinal phenotype. The second model is wild type (C57BL6) mice injected in the eye, intravitreal with Hcy-thiolactone (200 µM), the active metabolite of Hcy. In vitro, human retinal endothelial cells (HRECs) and RPE cells treated with/without Hcy were used [29,30]. Both in vivo models were subjected to functional studies using optical coherence tomography (OCT) and fluorescein angiography (FA) to evaluate retinal morphology and BRB integrity in living mice. Our data revealed retinal vascular leakage appeared as diffused hyperfluorescence on FA examination and marked disruption of the morphology of the retina, inner retinal, and choroidal neovascularization on OCT examination of the mice with HHcy compared to normal mice [27,28,29]. 

Moreover, functional assays were performed to assess HHcy-induced disruption of barrier function in HRECs and RPE cells. Real-time analyses of transendothelial electric resistance (TER) and FITC dextran flux were used to asses HRECs and RPE cells barrier function. Cells treated with different concentrations of Hcy showed significant reductions in TER and significant increase in permeability to FITC-dextran in a dose-dependent manner as indicated by increasing diffusive flux (Po) for FITC-dextran compared to vehicle-treated cells [29,30]. 

## 4. Hyperhomocysteinemia and Retinal Diseases

Retina is a part of the central nervous system that has a neurovascular nature. Recently, special attention has grown to study elevated Hcy level in relation to retinal diseases. Several clinical studies suggested its involvement to both neuronal and vascular diseases of the retina such as; central retinal venous [31,32], retinal artery occlusion [33,34], glaucoma [35], and corneal pathologies and cataracts [36,37,38]. Furthermore, elevated Hcy has been suggested as a risk factor and a biomarker for the most common retinal diseases resulting in loss of vision worldwide such as DR [39,40,41,42,43,44] and AMD [45,46,47,48].

## 5. Hyperhomocysteinemia and Age-Related Macular Degeneration

Age-related macular degeneration (AMD) is the most common and serious sight-threatening complication in people over age 60 [49]. AMD is a widespread public health problem that has considerable impact on the patient’s quality of life, the health care system, and society. In the United States, approximately 1.75 million persons have advanced AMD with associated vision loss and this number is expected to grow to almost 3 million by 2020 [50]. AMD has two clinical stages: Early AMD, evidenced by drusen and pigmentary change, and usually associated with normal vision; and late or sight threatening AMD, associated with a decrease in central vision. Late stage AMD has two forms: atrophic (or dry) AMD, characterized by geographic atrophy in which cells in the epithelial lining of the retina start to degenerate, resulting in regional loss of RPE cells, followed by death of the overlying photoreceptors [51] and exudative (or wet) AMD, characterized by choroidal neovascularization (CNV). 

The disruption of RPE function and the degeneration of photoreceptors are fundamental features of AMD. Recently, human studies have reported elevated plasma Hcy in patients with AMD and highlighted a strong association between HHcy and the development of AMD [48].

In our previous study [29], we investigated whether elevated Hcy acts directly on RPE structure and function leading to the development of AMD-like features. In vivo mouse model of excess Hcy (because of *cbs* deficiency) and in vitro human retinal pigment epithelial cell line (ARPE-19) model were used. We found that retinas of cbs deficient mice (*cbs*^+/−^ and *cbs*^+/−^) showed an uneven appearance and changes similar to those that occur in AMD. These features include decreased retinal thickness, atrophy at the level of the photoreceptors, subretinal fluid accumulation, hyporeflective subretinal lucency, thickened basal laminar membrane, separation of RPE, and increased thickness in the area of the choroid suggesting a possible CNV. Also, mice injected intravitreal with Hcy showed the same features of AMD, hyporeflective subretinal lucency, subretinal fluid and CNV compared to vehicle-injected controls. CNV formation was confirmed in retinal frozen sections by staining with the blood vessel marker, isolectin-B4.

Moreover, we investigated the ultrastructural morphology of the RPE cells in *cbs*^+/+^ and *cbs*^+/−^ mouse retinas using electron microscopy. Wild type *cbs*^+/+^ mouse retina revealed normal cuboidal RPE cells with very long sheet-like apical microvilli that project and interact with the photoreceptor outer segments and highly folded basal surface that interact with the underlying Bruch’s membrane separating the RPE from the choriocapillaries. In contrast, the *cbs*^+/−^ mouse retina showed abnormal RPE structure with less pigmentation and accumulation of pigmented particles in the lower part of RPE instead of the apical part, lost apical microvilli, and disturbed RPE-photoreceptor relation as well as thickened Bruch’s membrane. Furthermore, *cbs*^+/−^ mouse retinas showed accumulation of extracellular material between Bruch’s membrane and the RPE suggesting a possible drusen formation. Disruption of the Bruch’s membrane was also observed [29].

From our study we concluded that Hcy have deleterious effects on RPE, therefore Hcy-clearing mechanisms could be promising treatments for AMD.

## 6. Hyperhomocysteinemia and Diabetic Retinopathy

Diabetes mellitus is a microangiopathic atherosclerotic disease that affects the capillary bed in several organs, mainly the retina, kidneys, and peripheral nervous system. Chronic hyperglycemia is the major cause of DR [52,53]. Other factors that can affect the development and progression of DR include puberty [54], hypertension [55], and pregnancy [56]. As the severity of DR varies among diabetic patients with similar duration and control of the disease so perhaps there are other risk factors involved in the pathogenesis of DR. 

Since diabetes is a microvascular occlusive disease, an adjuvant risk factor contributing to a hypercoagulability state, such as increased levels of plasma Hcy, may accelerate or aggravate the development or progression of DR. Hcy is toxic to the vascular endothelium and therefore induces thrombosis, and thus may play a role in aggravating the hypoxic state such as that seen in DR by further closure of the capillary bed [57].

Recently, studying the role of Hcy in DM has gained increasing attention. Several studies stated that Hcy level is increased in the plasma of type 1 diabetes mellitus (T1DM) patients compared to non-diabetic controls [58,59]. Also, plasma Hcy level showed significant elevations in T1DM and type 2 diabetes (T2DM) patients with DR compared to patients without any complications [60], suggesting that increased Hcy levels during DM contributes to the development of retinopathy. Several clinical studies suggested an association between elevated serum Hcy levels and the risk of DR [43,44,61]. There is correlation between HHcy and diabetes-induced microangiopathies such as diabetic nephropathy, retinopathy, and macular edema [62,63,64]. Elevated retinal Hcy level induced mitochondrial damage in retinal microvasculature from human donors with established DR and this was accompanied by compromised Hcy metabolism [65]. Moreover, patients with T2DM showed HHcy accompanied by increased macular thickness and volume prior to the development of macular edema, suggesting HHcy as a biomarker for macular edema in diabetic patients [40]. Therefore, Hcy could be used as a marker and may be as a future therapeutic target for DR via enhancing Hcy clearance by pharmacological or genetic manipulations which could be a preventive/therapeutic strategy for DR.

In our previous study, we measured Hcy and CBS levels in human and different animal models of T1DM and T2DM. Our study demonstrated a significant increase in Hcy concentration and decrease in CBS level in the serum and retina of human and different animal models of diabetes [66]. Increased Hcy levels may occur because of several reasons as we mentioned before, these reasons include deficiency of folic acid and vitamin B12 or deficiency of any enzymes needed for Hcy metabolism such as CBS enzyme [67,68]. Additionally, Ratnam et al. reported that insulin plays a role in controlling Hcy metabolism. Therefore, impaired insulin levels in diabetes may lead to increased Hcy level [69]. 

Vasculopathies linked to HHcy could be a part of the mechanism by which Hcy causes DR. These vasculopathies include endothelial dysfunction, vessel wall malformations, loss of extracellular matrix collagen, and disruption of the blood–retinal barrier (BRB) [70]. Moreover, our previous work reported that excess Hcy induced retinal ischemia and neovascularization via increasing vascular endothelial growth factor (VEGF) level in retina [27,28,29]. Also, we showed that excess Hcy leads to activation of endoplasmic reticulum (ER) stress and oxidative stress [30,71].

Additionally, Hcy induces the death of retinal ganglion cells [72,73] leading to retinal neurodegeneration. Hcy has been reported to induce apoptosis in retinal ganglion cells and induced ganglion cell loss [74,75,76]. Several studies suggested that activation of NMDA receptors could be a possible mechanism of HHcy-induced retinal ganglion cell death [76,77,78]. Other studies suggested that HHcy could produce its toxic effect via the activation of inflammatory and oxidative stress mechanisms causing activation of mitogen-activated protein kinases (MAPK), macrophage infiltration, and enhanced pro-inflammatory cytokines production [79]. Srivastav et al. showed that Hcy level was correlated with the decrease in retinal nerve fiber layer thickness in diabetic patients [44]. 

## 7. Possible Mechanisms of Homocysteine-Induced BRB Dysfunction

Different mechanisms have been proposed to explain Hcy-induced visual dysfunction, including ischemic vascular dysregulation [80], retinal ganglion cells apoptosis [81], oxidative stress [82] alteration of inflammatory mediators, and extracellular matrix remodeling [83]. Observations in patients with homocystinuria and experimental models of HHcy have proposed several mechanisms for Hcy–induced vascular diseases. Hcy has detrimental effects on endothelial cells, vascular smooth muscle cells, platelets, coagulation factors, blood lipids, and nitric oxide [81,82]. Animal models studies have demonstrated that high levels of Hcy lead to increased oxidant stress and impaired endothelial function which subsequently led to atherosclerosis [80,83]. Hcy toxic effect on vascular endothelial cells results in proliferation of smooth-muscle cells, oxidation of low-density lipoprotein, and increase in collagen synthesis and pro-coagulant activity [84]. Also, case-control and cross-sectional studies have showed association between Hcy plasma level and the extent of carotid, coronary, and peripheral vascular disease [85]. Our previous studies assessed the direct effect of Hcy on the different components of BRB, including retinal endothelial cells, RPE cells, glia cells, and pericytes. Furthermore, our studies investigated the underlying mechanisms involved in HHcy-induced vascular and BRB dysfunction. 

**Figure 4:**
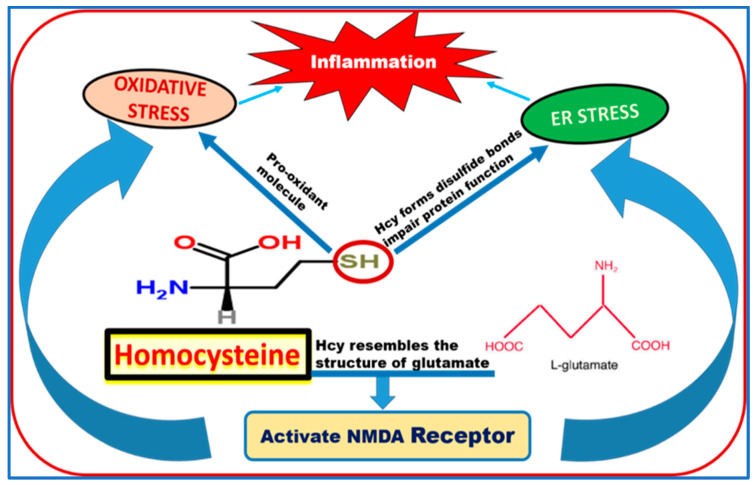
Suggested mechanisms of homocysteine induced harmful effects on retina and blood retinal barrier.

We suggested many mediators and pathways participating in Hcy-induced harmful effects on retinal vasculature and BRB dysfunction. As shown in (Figure 4), Hcy sulfhydryl (-SH) group could act as pro-oxidant molecule that increases the oxidative stress. Also, SH group of Hcy forms disulfide bonds which impair protein function and lead to endoplasmic reticulum (ER) stress. In our previous published work, we reported that Hcy induced BRB via the activation of oxidative stress and ER stress [30,86]. Both oxidative stress and ER stress subsequently could result in the activation of several inflammatory pathways [87]. ER is involved in protein synthesis, maturation and transport [88], and regulates cell energy metabolism, redox status, inflammation, and cell survival through the unfolded protein response (UPR) [89,90]. We reported increased ER stress genes and proteins of BiP/GRP78 and PERK in retinas of *cbs*^−/−^ mice compared to wild type *cbs*^+/+^ mice. We also found increased immunostaining of CCAAT-enhancer-binding protein homologous protein (CHOP), a transcription factor involved in ER stress-induced apoptosis in retinal nerve fiber layer, ganglion cells, and inner nuclear layer accompanied by elevated levels of apoptotic markers (PARP and cleaved caspase-3) in the retinas of *cbs*^−/−^ mice, suggesting implication of ER stress/apoptosis in the neuro-vasculopathy associated with the HHcy-linked retinal disease [71]. 

Recent studies reported that increased ER stress leads to retinal vascular hyperpermeability via the NF-κB inflammatory signaling pathway [91,92,93]. Induction of ER stress in mouse eyes increased retinal expression of TNF-α and VEGF, and vascular leakage, while inhibition of ER stress reduced these changes in diabetic and ischemic retinas [91,93,94,95] suggesting that ER stress plays a role in the breakdown of the BRB in retinal diseases associated with vascular hyperpermeability.

Our studies suggested oxidative stress is a potential player in Hcy-induced retinal endothelial hyperpermeability and dysfunction [21]. We found that elevated Hcy levels induced oxidative stress in retinas of *cbs*^−/−^ mice. Moreover, retinas of CBS deficient mice showed decreased levels of the antioxidant markers such as superoxide dismutase (SOD) and glutathione peroxidase (GPX) and the main antioxidant in the retina glutathione (GSH). In vitro, Hcy-treated HRECs showed a significant decrease in GSH and a dose-dependent increase in ROS levels. Interestingly, the Hcy-induced increase of FITC dextran leakage in HRECs was abolished by co-treatment with the antioxidant *N*-acetyl-cysteine (NAC). 

A large body of evidence has linked Hcy to epigenetic modification [96,97]. Global DNA methylation status accompanied by HHcy was reported in patients with vascular diseases [98] and coronary artery disease [99]. HHcy results in gene hypermethylation, gene silencing, altered expression of histone modification genes and miRNA. These epigenetic modifications play crucial role in Hcy-induced stroke [100]. In this context, we reported HHcy-induced epigenetic modifications as potential mechanisms of blood retinal barrier BRB dysfunction. This was evidenced by increased activity of histone deacetylases (HDAC) and DNA methyl transferase (MTT) in vivo in *cbs* mice retinas, and in vitro in HRECs and ARPE-19 treated with Hcy. Further, inhibition of DNA methylation and histone deacetylation attenuated HHcy-induced BRB dysfunction. Moreover, miRNA profiling detected differential expression of 127 miRNAs in *cbs*^+/–^ and 39 miRNAs in *cbs*^–/–^ mice retinas compared to wild type mice retinas. Ingenuity pathway analysis showed that these differentially expressed miRNAs are involved in various metabolic pathways including oxidative stress, ER stress, autophagy, tight junctions signaling, inflammation, and angiogenesis. Interestingly, common differentially expressed miRNA were found in *cbs*^–/–^, diabetic mouse retina, and exosomes released from Hcy-treated ARPE. Hence, our findings highlight epigenetic modifications as possible mechanisms in HHcy-induced BRB dysfunction [86].

Recently, our studies have proved that activation of inflammatory signaling is implicated in HHcy-induced BRB dysfunction. In our published paper, we showed that Hcy activates microglia in the retina and results in the activation of NF-kB, a transcription factor that controls the expression of various inflammatory cytokines in HRECs and RPE. HHcy activates IL-1β and TNF-α in retina. Therefore, our results demonstrated that retinal inflammation can play a significant role in the development and progression of DR and AMD [78].

Moreover, we noticed the similarity between Hcy structure and L-glutamate structure; therefore, currently we are studying the ability of Hcy to activate the glutamate receptor *N*-methyl-d-aspartate receptor (NMDAR) as one of Hcy mechanisms of action in retinal diseases. NMDAR, a subtype of ion-gated glutamate channel receptor, consists of 7 subunits (NR1, NR2A–D, NR3A, and B). The expression of NMDAR subunits is not limited to brain neurons; they are also present in astrocytes, osteoblasts and osteoclasts, peripheral neurons [101,102,103], and endothelial cells [104,105,106]. NMDAR is a receptor for Hcy in neurons [107]. Activation of NMDAR in cerebral endothelial cells by Hcy results in hyperpermeability by disrupting TJs [70,108,109]. The NMDAR antagonist memantine prevents BBB permeability in experimental models of multiple sclerosis [110] and reverses attenuating effects of HHcy on adherens junctions (VEC/β-catenin) and TJs (claudin-5) in brains of *cbs*^+/−^ mice [70]. Thus, NMDAR could affect endothelium by mediating intracellular signal transduction in response to increases in Hcy concentration in the blood. We have evidence that Hcy-induced neurotoxicity is mediated via activation of the NMDAR [77], resulting in cytoplasmic calcium influx, activation of the inflammatory pathway and cellular apoptosis [111,112,113]. The hybridization signals for NR2A and NR2B mRNA were significantly elevated in the dorsal horn of diabetic rats [114]. Alterations of the NMDAR, damage to ganglion cell mitochondria [77], and marked alterations of retinal function were observed in *cbs*^−/−^ and *cbs*^+/−^ mice [95]. Previous studies and our current studies suggest that activation of NMDAR contributes to HHcy-induced breakdown of the BRB.

## 8. Hyperhomocysteinemia Is a Risk Factor for Neurological Diseases

HHcy has been shown to be associated with stroke, dementia, Alzheimer, and cerebral small-vessel disease [115]. Patients with acute stroke and HHcy are at an increased risk for early neurological deterioration [116]. Clinical studies have demonstrated that HHcy is associated with the shift from being cognitively healthy to developing dementia [117,118] and that HHcy plays a role in the decline of cognitive performance in normal elderly subjects and in Alzheimer patients. Also, several clinical studies have illustrated that Hcy is involved in the pathogenesis of Parkinson’s disease [119]. Licking et al. correlated HHcy with aspects of cognitive dysfunction in Parkinson’s disease [120]. Hcy increases the liability of dopaminergic neurons to damage and accelerates the progression of Parkinson’s disease [121]. In our published work [87], we reported that HHcy activates microglia in the brain and IL-1β and TNF-α in hippocampus area of the brain. Our data suggest that HHcy-induced inflammation could play a role in BBB dysfunction and the pathogenesis of Alzheimer disease.

Hcy-mediated vasculopathies are accompanied with endothelial dysfunction, vessel wall malformations, loss of extracellular matrix collagen, and loosening of the BBB [122,123]. HHcy increases BBB permeability which is a key factor in initiating stroke and cerebral small-vessel disease [124,125].

BBB is the barrier at the interface between the blood and the brain which prevents the movement of the undesired molecules. The BBB is formed by intercellular junctions that selectively transport molecules either into or out of the brain. BBB permeability play a key role in the onset of neurologic and neurovascular diseases [126]. Adherens junction and tight junction proteins minimize the diffusion of molecules at the BBB. These proteins include vascular-endothelial cadherin (VE-cadherin; VEC) and claudin-5 [70,127]. Hcy binds to NMDAR which is a glutamate receptor in neurons [78]. NMDAR is present in cerebral endothelium and its activation causes disruption of tight junctions and increasing permeability. Therefore, NMDAR is a rational target for therapeutic intervention in HHcy in the context of BBB disruption [109,128].

In conclusion, there is ample evidence that disturbed Hcy metabolism triggers critical pathways associated with various neurological and retinal diseases. Moreover, it is clear that elevated Hcy level induces a diverse set of pathological effects on BBB and BRB. Therefore, the key molecular targets of Hcy need to be fully defined. Besides, it remains to be elucidated whether HHcy is a causative factor or marker of damage. Further, development of drugs or interventions for clearance of Hcy may hold promise for management of diseases related to dysfunction of BBB and BRB.
